# miR-92a-2-5p Regulates the Proliferation and Differentiation of ASD-Derived Neural Progenitor Cells

**DOI:** 10.3390/cimb44060166

**Published:** 2022-05-24

**Authors:** Wenting Zhuang, Hui Liu, Zhize He, Jielan Ju, Qiuxia Gao, Zhiyan Shan, Lei Lei

**Affiliations:** Department of Histology and Embryology, Harbin Medical University, Harbin 150000, China; 201901016@hrbmu.edu.cn (W.Z.); liuhui@sdfmu.edu.cn (H.L.); hezhizelucky@163.com (Z.H.); m17860506681@163.com (J.J.); gqx479717@163.com (Q.G.)

**Keywords:** miR-92a-2-5p, neural precursor cells, proliferation, differentiation, ASD, DLG3

## Abstract

Autism spectrum disorder (ASD) is a group of complex neurodevelopmental disorders with abnormal behavior. However, the pathogenesis of ASD remains to be clarified. It has been demonstrated that miRNAs are essential regulators of ASD. However, it is still unclear how miR-92a-2-5p acts on the developing brain and the cell types directly. In this study, we used neural progenitor cells (NPCs) derived from ASD-hiPSCs as well as from neurotypical controls to examine the effects of miR-92a-2-5p on ASD-NPCs proliferation and neuronal differentiation, and whether miR-92a-2-5p could interact with genetic risk factor, *DLG3* for ASD. We observed that miR-92a-2-5p upregulated in ASD-NPCs results in decreased proliferation and neuronal differentiation. Inhibition of miR-92a-2-5p could promote proliferation and neuronal differentiation of ASD-NPCs. *DLG3* was negatively regulated by miR-92a-2-5p in NPCs. Our results suggest that miR-92a-2-5p is a strong risk factor for ASD and potentially contributes to neuropsychiatric disorders.

## 1. Introduction

Autism spectrum disorder (ASD) is a heritable, highly pervasive neurodevelopmental disorder characterized by impaired social communication and interaction, as well as repetitive and stereotypical behavior [1]. The incidence of ASD at 8 years old increased from 1 in 54 in 2016 to 1 in 44 children in 2018 in the United States. Unfortunately, the morbidity of ASD is higher than expected, and the alarming increase in ASD reported over the last two decades also highlights an important public health issue [2,3].

A variety of factors contribute to the pathogenesis of ASD, in which epigenetics might play an important role [4,5]. MiRNAs are a class of small (approximately 21–25 nucleotides), noncoding RNAs that can modulate cellular mRNA and protein levels through partial sequence complementation, resulting in mRNA degradation or translation repression [6,7]. About fifty percent of miRNAs express and distribute in distinct regions in the mammalian brain. They act important roles in neuroinflammation [8], neural stem cell proliferation, differentiation [9], and neurogenesis [10]; additionally, they take part in several neurodegenerative diseases, such as schizophrenia, Alzheimer’s disease, and ASD [11,12,13,14]. For instance, loss of miR-146a impairs the differentiation of radial glial cells, neurogenesis process, and neurite extension, which can cause severe hippocampal-dependent learning and memory impairments. miR-873 can affect the morphology of neurons by regulating the ASD risk genes *ARID1B* and *SHANK3*. Altered miRNA expression has been found in the brain and peripheral blood of ASD patients, and miRNA may act as an important factor in the progression of ASD [15,16].

Previously published works have shown the critical roles of miR-92a in neuronal neurite outgrowth, neuronal differentiation, and GABAergic neuron maturation [17,18,19]. One such miRNA called miR-92a-2-5p shares the same seed sequence with miR-92a, which suggests that their functions are highly correlated. Our previous study revealed that miR-92a-2-5p levels were decreased in astrocytes derived from ASD-human induced pluripotent stem cells (hiPSCs), which indicated miR-92a-2-5p might also play a vital role in ASD. However, this hypothesis remains to be confirmed. In the present study, we demonstrated that NPCs derived from ASD-hiPSCs exhibit decreased proliferation with delayed S phase progression, and elevated miR-92a-2-5p levels were also found in ASD-NPCs. Moreover, neurons derived from ASD-NPCs exhibit earlier differentiation deficiency and a reduction in inhibitory neurons. Inhibition of miR-92a-2-5p could promote proliferation and differentiation of ASD-NPCs. *DLG3* might be the target gene of miR-92a-2-5p.

Taken together, our data indicate that miR-92a-2-5p is an important regulator of proliferation and neuronal differentiation of NPCs derived from ASD-hiPSCs; the data also suggest that aberrant miR-92a-2-5p expression may account for the pathophysiology of autism.

## 2. Materials and Methods

### 2.1. NPC Culture and Transfection

The samples were collected from the foreskin tissue of ASD patients and children without ASD (Supplementary Table S1). hiPSCs were established using Cytotune Reprogramming kit (A16517, Thermo, Waltham, MA, USA) and plated onto Matrigel (354277, Corning, NY, USA) coated plates in Neural Progenitor Medium (05833, STEMCELL), and then were used to induce NPCs. NPCs were passaged at a ratio of 1:3 every three days using Accutase (7920, STEMCELL).

NPCs were transfected with miR-92a-2-5p mimic or mimic negative control at a final concentration of 50 nM, or inhibitor and inhibitor negative control at a final concentration of 100 nM, using Lipofectamine 3000 (Thermo, L3000008) for 8 h according to the manufacturer’s protocol. RNA and protein expression were determined at 24 h and 48 h after incubation.

### 2.2. NPC Proliferation Assay

EdU staining was conducted to detect the proliferation of NPCs using the BeyoClick™ EdU Cell Proliferation Kit with Alexa Fluor 594 (C00788L, Beyotime, Shanghai, China) following the manufacturer’s instructions. NPCs were seeded in 6-well plates (6 × 10^5^ cells per well) and cultured with fresh NPC medium supplemented with 10 µM EdU for 2 h. After washing with DPBS, cells were fixed with 4% PFA solution at 4 °C for 30 min. Cells were incubated with 4′,6-diamidino-2-phenylindole (DAPI) for 5 min to stain nuclei. The images were collected with an inverted microscope (Nikon), and GraphPad 8 was used to analyze positive cells.

Cell counting kit-8 (CCK-8) (Transgen, FC101) was used to measure the cell proliferation. Cells were seeded in a microplate (2 × 10^3^ cells per well), and after 24 h, 10 μL of CCK-8 reagent was added. The cells were incubated for an additional 2 h at 37 °C, and then absorbance was measured with a microplate reader (ELX808, INFINITE M, Männedorf, Switzerland) at 450 nm.

The cell cycle was analyzed by flow cytometry (Beyotime, C1052) following the manufacturer’s instructions. Cells were fixed with 70% prechilled ethanol at 4°C overnight. Propidium iodide (PI) was added and the cells were stored at 37 °C for 30 min. Then the cells were measured with a flow cytometer (FACS Calibur, BD, Franklin Lakes, NJ, USA) and each cell cycle phase was analyzed by FlowJo software.

### 2.3. Neuronal Differentiation

NPCs were seeded in a poly-L-lysine (P5899, Sigma, St. Louis, MO, USA) and laminin (L2020, Sigma) coated 24-well plate (1 × 10^5^ cells per well) with Neuronal Medium (1521, ScienCell, Carlsbad, CA, USA). After 10, 20, and 30 days, the immature, mature, and inhibitory neurons were observed by immunofluorescence.

### 2.4. Immunofluorescence

For immunostaining, cultured NPCs were collected and fixed in 4% PFA for 30 min at 4 °C. Cells were blocked with 2% BSA and 0.2% Triton X-100 in 1× PBS at room temperature for 30 min. The following primary antibodies were used for immunostaining: anti-Ki67 (1:200, 556003, BD, NJ, USA), anti-Tuj1 (1:200, #8173, CST, MA, USA), anti-MAP2 (1:200, 17490-1-AP, Proteintech, IL, USA), and anti-VGAT (1:200, 14471-1-AP, Proteintech, IL, USA). After washing with DPBS, cells were incubated with secondary antibodies for 1 h at room temperature. DAPI was used to label cell nuclei. The images were collected with a confocal microscope (Nikon), and GraphPad 8 was used to analyze positive cells.

### 2.5. RT-qPCR Analysis

Total RNA was isolated from tissues and cells using the miRNeasy Mini kit (Qiagen, 74104) according to the manufacturer’s protocol. RT-qPCR was performed using TransStart^®^ Top Green qPCR SuperMix (Transgen, AQ131-01, Beijing, China) and a CFX96 Real-time System (Bio-Rad, Irvine, CA, USA). U6 and GAPDH were used for normalization of miRNA and mRNA expression, respectively. RT-qPCR was carried out with three replications. Primer sequences are described in Table S2.

### 2.6. Western Blot

Cultured NPCs was lysed in RIPA buffer containing PMSF (100×) for 30 min at 4 °C. Lysates were centrifuged for 30 min at 12,000 rpm at 4 °C and the supernatants were collected. The protein concentration was measured by BCA assay and supernatants were mixed with 6× loading buffer. Proteins were separated by 10% SDS-PAGE (15 μg protein per lane) and transferred onto PVDF membranes. The following primary antibodies were used: anti-CDK2 (1:4000, 10122-1-AP, Proteintech, Rosemont, IL, USA), anti-GAPDH (1:10,000, KC-5G4, KangChen Bio-tech, Shanghai, China), anti-cyclin A2 (1:1000, AF0142, Affinity, Melbourne, Australia), and anti-PCNA (1:4000, 10205-2-AP, Proteintech). After incubation with HRP-conjugated secondary antibody (ab6721, Abcam, Cambridge, UK), the protein bands were visualized using enhanced chemiluminescence reagent (35477, Thermo, MA, USA). The images were collected with a ChemiDoc Imaging System Bio-Rad, Irvine, CA, USA), and gray values were analyzed using ImageJ software (National Institutes of Health, Bethesda, MD, USA).

### 2.7. Statistical Analyses

The experimental data were conducted on at least 3 biological replicates, and statistical analyses were performed in GraphPad 8 (GraphPad Software Inc., La Jolla, CA, USA). Differences between groups were analyzed using Student’s t-test. Data are presented as mean ± SD. Statistical significance was set at *p* < 0.05.

## 3. Results

### 3.1. iPSC Derived NPCs from ASD-hiPSCs Exhibit Decreased Proliferation and Delayed Cell Cycle Progression

Previous studies showed that neuronal differentiation of iPSCs from ASD patients could recapitulate fetal brain development, so we chose NPCs derived from CON and ASD hiPSCs to model the aberrant developmental stage [20]. To explore the potential neuronal development deficits, we investigated the proliferation capacity, apoptosis, and cell cycle of ASD-NPCs.

**Figure 1:**
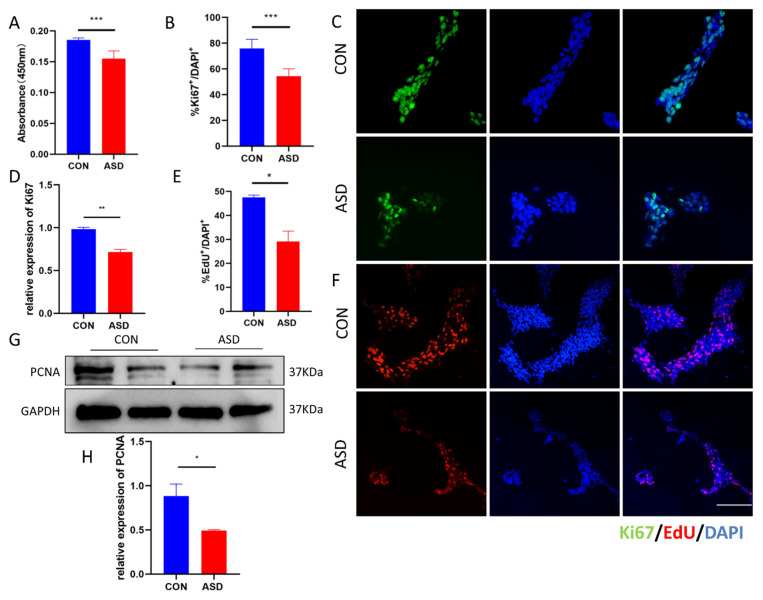
Impaired proliferation of ASD-NPCs. (**A**) Cell viability of CON and ASD-NPCs was assessed by CCK-8 assay. (**B**,**C**) CON and ASD-NPCs were immunostained with anti-Ki67 (green). DAPI (blue) was used to stain nuclei. The percentage of Ki67-labeled cells was calculated. (**D**) Relative expression of Ki67 in CON and ASD-NPCs as determined by RT-qPCR. (**E**,**F**) CON and ASD-NPCs were stained with DAPI (blue) and EdU (red). The percentage of EdU-labeled cells was calculated. (**G**,**H**) Relative expression of PCNA in CON and ASD-NPCs as determined by western blot. All data represent mean ± SEM; scale bar: 100 μm; *** *p* < 0.001, ** *p* < 0.01, * *p* < 0.05.

First, we conducted CCK-8 assays and found that ASD-NPCs grew slowly compared with CON-NPCs (Figure 1A). Quantification of Ki67^+^ cells indicated a decreased percentage of proliferating cells in ASD-NPCs, and RT-qPCR analysis showed that expression of Ki67 was also decreased (Figure 1B–D). In addition, the proportion of EdU-positive cells was reduced in ASD-NPCs (Figure 1E,F), and expression of the proliferation marker PCNA was decreased in ASD-NPCs, as determined by western blot (Figure 1G,H). These results demonstrated that the proliferative capacity of ASD-NPCs was impaired.

**Figure 2:**
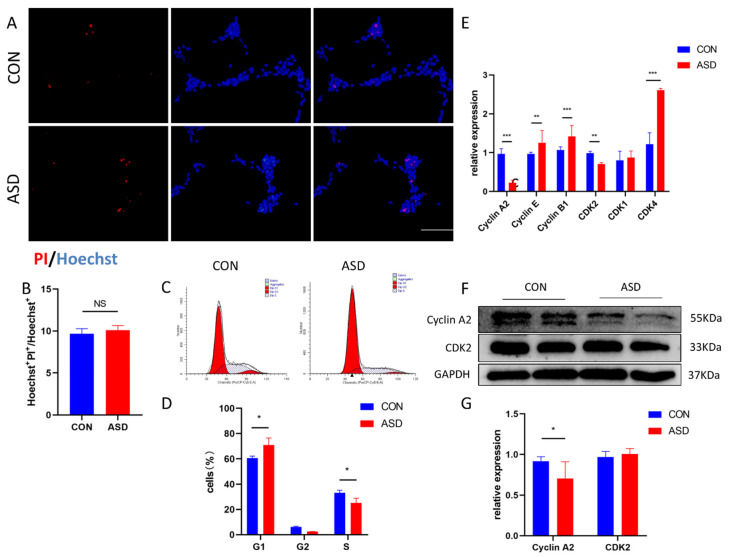
Abnormal cell cycle of ASD-NPCs, with no difference in apoptosis. (**A**,**B**) CON and ASD-NPCs were stained with Hoechst (blue) and PI (red). The percentage of PI-labeled cells was calculated. (**C**,**D**) FCM analyses of the cell cycle of NPCs showing the percentages of cells at different cell cycle phases. (**E**–**G**) Relative expression of cell cycle-related genes of CON and ASD-NPCs as determined by RT-qPCR and western blot. All data represent mean ± SEM; scale bar: 100 μm; *** *p* < 0.001, ** *p* < 0.01, * *p* < 0.05.

Second, Hoechst 33342 and PI staining assays were used to analyze apoptosis (Figure 2A). No significant difference was observed between CON and ASD-NPCs (Figure 2B). Cell cycle analysis revealed that the proportion of cells in the S phase was decreased in ASD-NPCs (Figure 2C). Since the protein kinase complexes composed of cyclins and cyclin-dependent kinases (CDKs) determine the progression of cells through the cell cycle, we detected the expression of some key factors in the cell cycle by RT-qPCR and western blot. As shown in Figure 2D–F, Cyclin A2 expression was decreased in ASD-NPCs. Collectively, these data indicated that ASD-derived NPCs displayed decreased proliferation with delayed S phase progression.

### 3.2. iPSC Derived NPCs from ASD-hiPSCs Exhibit Decreased Neuronal Differentiation

**Figure 3:**
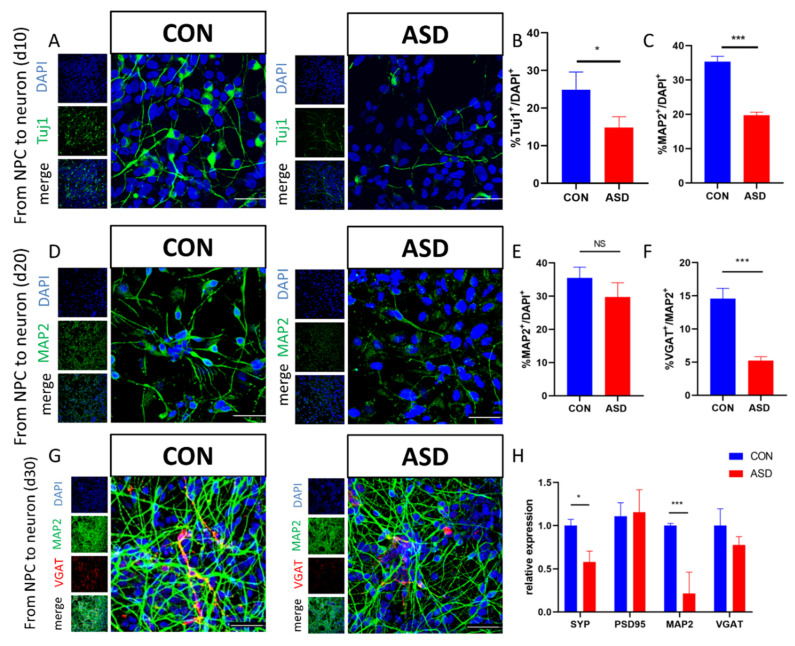
Impaired differentiation of ASD-NPCs. (**A**,**B**) Representative images of TUJ1 (green)-expressing neurons derived from CON and ASD-NPCs at day 10. DAPI (blue) was used to stain nuclei. The percentage of TUJ1-labeled cells was calculated. (**C**,**D**) Representative images of MAP2 (green)-expressing neurons derived from CON and ASD-NPCs at day 20. The percentage of MAP2-labeled cells was calculated. (**E**–**G**) Representative images of MAP2 (green) and VGAT (red)-expressing neurons derived from CON and ASD-NPCs at day 30. The percentage of MAP2- and VGAT-labeled cells was calculated. (**H**) Relative mRNA expression of MAP2, PSD95, SYP, and VGAT in control and ASD-NPCs at day 30 as determined by RT-qPCR. All data represent mean ± SEM; scale bar: 50 μm; *** *p* < 0.001, * *p* < 0.05.

To detect changes in neuronal differentiation of NPCs between the ASD and CON groups, ASD-and CON-NPCs were cultured in neuronal medium for 10, 20, 30 days, and then the expression levels of typical markers for early (Tuj1) and mature (MAP2) neural cells and inhibitory neurons (VGAT) were measured by immunofluorescence. The numbers of Tuj1 and MAP2 positive cells were decreased at 10 and 20 days post-differentiation, indicating that the early stage of neuronal differentiation in ASD-NPCs was impaired (Figure 3A–D). Interestingly, no significant difference in MAP2^+^ cells was observed at 30 days after differentiation, which means the early developmental defects were compensated for at the late stage of neuronal differentiation (Figure 3E,G). Evidence from the literature suggested that an imbalance in excitatory versus inhibitory signals exists in developing ASD [21]. Therefore, we tested whether GABAergic cell fate determination was affected in ASD-NPCs. Indeed, a reduction in the percentage of VGAT^+^ (inhibitory neuron marker) in ASD neurons compared with CON was observed (Figure 3F,G). VGAT mRNA expression was slightly lower in ASD-NPCs, but this difference was not significant (Figure 3H). Next, using RT-qPCR methods, we evaluated the levels of pre-and postsynaptic related proteins PSD95 and SYP (synaptophysin). Our results showed that ASD-NPCs exhibit the losses of SYP. These results indicated that ASD-NPC derived neurons exhibit earlier differentiation deficiency, a reduction in inhibitory neurons, and synapse loss.

### 3.3. Knockdown of miR-92a-2-5p Promotes Proliferation and Neuronal Differentiation of ASD-NPCs

**Figure 4:**
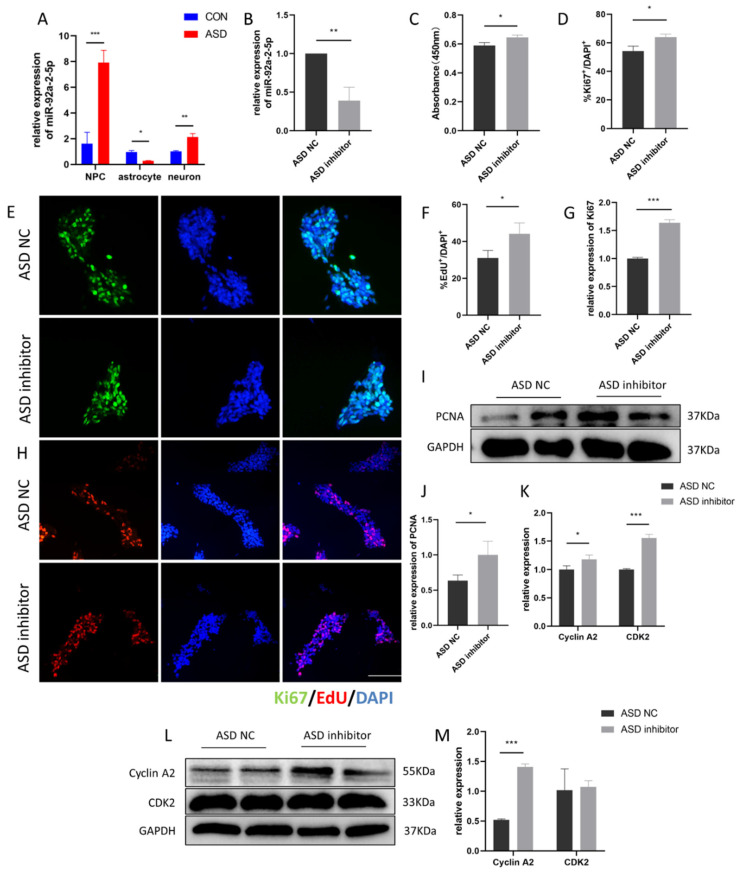
miR-92a-2-5p modulates the proliferation of ASD-NPCs. (**A**) Relative expression of miR-92a-2-5p in ASD-NPCs, astrocytes, and neurons, as determined by RT-qPCR. (**B**) Verification of decreased expression of miR-92a-2-5p 24 h after transfection with miR-92a-2-5p inhibitor in ASD-NPCs using RT-qPCR. (**C**) Cell viability was assessed via CCK-8 assays in ASD NC and ASD inhibitor. (**D**,**E**) ASD NCs and ASD inhibitor NPCs were immunostained with anti-Ki67 (green). DAPI (blue) was used to stain nuclei. The percentage of Ki67-labeled cells was calculated after transfection with miR-92a-2-5p inhibitor. (**F**) Relative mRNA expression of Ki67 in ASD NC and ASD-inhibitor NPCs determined by RT-qPCR. (**G**,**H**) ASD NC and ASD inhibitor NPCs were stained with DAPI (blue) and EdU (red). The percentage of EdU-labeled cells after transfection with miR-92a-2-5p inhibitor was calculated. (**I,J**) Relative expression of PCNA in ASD NC and ASD inhibitor NPCs determined by western blot. (**K**–**M**) Relative expression of Cyclin A2 and CDK2 of ASD NC and ASD inhibitor NPCs, as determined by RT-qPCR and western blot. All data represent mean ± SEM; scale bar: 100 μm; *** *p* < 0.001, ** *p* < 0.01, * *p* < 0.05.

Upregulation of miR-92a-2-5p could decrease proliferation of rat intestinal epithelial cells [22]. To identify the target genes of miR-92a-2-5p, we employed a bioinformatics approach using two miRNA target prediction tools—TargetScan and miRDB. GO analysis was used to reveal the biological processes in which these potential target genes are involved that are important for neuronal function, such as ion channel binding, nervous system development, and hippocampus development (Supplementary Figure S1). Moreover, we found that miR-92a-2-5p was highly expressed in NPCs and neurons, and its expression was lower in astrocytes of the ASD group (Figure 4A). According to these results, we hypothesized that upregulation of miR-92a-2-5p might give rise to abnormal proliferation and differentiation of ASD-NPCs. To examine whether treatment of NPCs with miR-92a-2-5p inhibitor alters the expression of miR-92a-2-5p, ASD-NPCswere cultured in NPC medium containing either vehicle or miR-92a-2-5p inhibitor for 24 h, and the relative expression of miR-92a-2-5p was determined by RT-qPCR. The expression of miR-92a-2-5p was downregulated, confirming the transfection was successful (Figure 4B).

First, the CCK-8 assay revealed that downregulation of miR-92a-2-5p promoted proliferation of ASD-NPCs (Figure 4C). We next sought to evaluate the effect of miR-92a-2-5p on the proliferation and cell cycle of ASD-NPCs. Quantification of Ki67^+^ and EdU^+^ cells indicated an increased percentage of proliferating cells in ASD-NPCs after miR-92a-2-5p inhibitor transfection (Figure 4D–F,H). Furthermore, we analyzed the expression of Ki67 and PCNA by RT-qPCR and western blot, which were increased in the ASD inhibitor group (Figure 4G,I,J). These results indicated that miR-92a-2-5p downregulation promotes proliferation of ASD-NPCs. Next, RT-qPCR and western blot were used to verify the expression of cell cycle genes. Cyclin A2 expression was increased, which indicated that the proportion of cells in the S phase was evaluated in the ASD inhibitor group (Figure 4K–M). Altogether, these results suggested that knockdown of miR-92a-2-5p in ASD-NPCs prolonged the S phase and promoted proliferation of ASD-NPCs.

**Figure 5:**
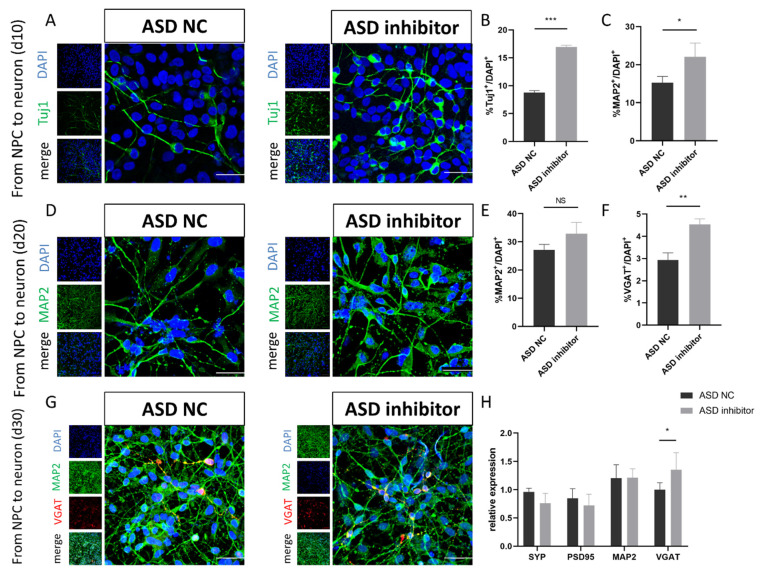
MiR-92a-2-5p modulates the differentiation of ASD-NPCs. (**A**,**B**) Representative images of TUJ1 (green)-expressing neurons derived from ASD NC and ASD inhibitor NPCs at day 10. DAPI (blue) was used to stain nuclei. The percentage of TUJ1-labeled cells was calculated. (**C**,**D**) Representative images of MAP2 (green)-expressing neurons derived from ASD NC and ASD inhibitor NPCs at day 20. The percentage of MAP2-labeled cells was calculated. (**E**–**G**) Representative images of MAP2- (green) and VGAT (red)-expressing neurons derived from ASD NC and ASD-inhibitor NPCs at day 30. The percentage of MAP2- and VGAT-labeled cells was calculated. (**H**) Relative mRNA expression of SYP, PSD95, MAP2, and VGAT in ASD NC and ASD inhibitor NPCs at day 30, as determined by RT-qPCR. All data represent mean ± SEM; scale bar: 50 μm; *** *p* < 0.001, ** *p* < 0.01, * *p* < 0.05.

Then, we sought to determine whether miR-92a-2-5p has any effect on the neuronal differentiation of ASD-NPCs. For this purpose, CON and ASD-NPCs were cultured in neuronal medium for 10, 20, 30 days and then the expression levels of typical markers for neurons, inhibitory neurons, and synapse were measured by immunofluorescence and RT-qPCR. At 10 and 20 days post-differentiation, the numbers of TUJ1^+^ and MAP2^+^ cells were increased in the ASD inhibitor group (Figure 5A–D). No significant difference in MAP2^+^ cells was observed at 30 days after differentiation transfected with miR-92a-2-5p inhibitor (Figure 5E,G). MiR-92a-2-5p inhibitor transfection during induction also rescued the decrease in inhibitory neurons and increased VGAT^+^ neurons and mRNA expression of VGAT, with no significant difference in SYP and PSD95 (Figure 5F–H). Together, these results indicated that downregulation of miR-92a-2-5p in ASD-NPCs could increase inhibitory neurons and restore the early differentiation deficiency, which suggested that miR-92a-2-5p might be a key regulatory miRNA in the neuronal differentiation of ASD-NPCs.

### 3.4. DLG3 Might Be a Target of miR-92a-2-5p

**Figure 6:**
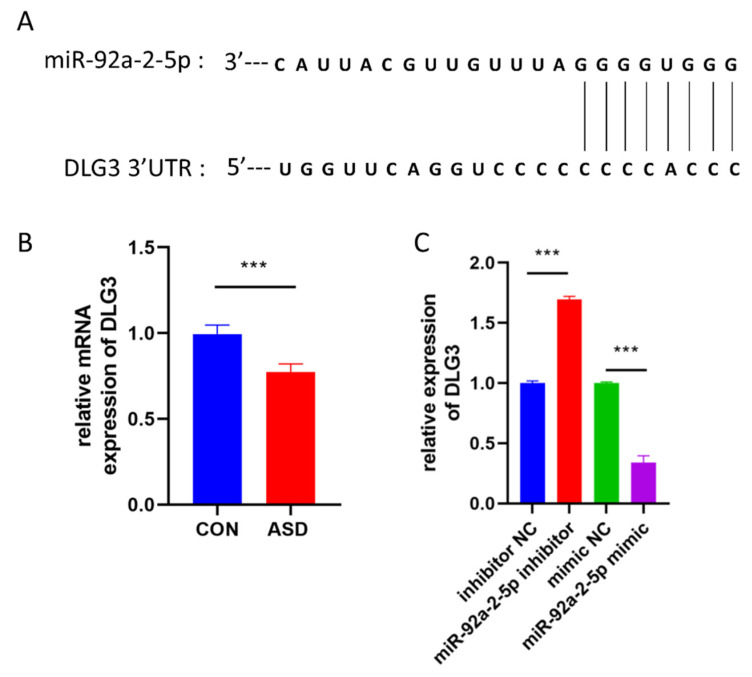
DLG3 might be a target gene of miR-92a-2-5p. (**A**) Putative target sites for miR-92a-2-5p in the 3′UTR of DLG3. (**B**) Relative mRNA expression of DLG3 in CON and ASD-NPCs, as determined by RT-qPCR. (**C**) Relative mRNA expression of DLG3 after transfection with miR-92a-2-5p inhibitor, mimic, and negative control in NPCs. All data represent mean ± SEM; *** *p* < 0.001.

miRNAs usually regulate the translation and degradation of target mRNAs by binding to their 3’UTR region [23]. To identify the potential target genes of miR-92a-2-5p, we used miRNA target prediction algorithms (TargetScan and miRDB). The 3’UTR of DLG3 mRNA contains a potential miR-92a-2-5p binding site (Figure 6A). Our RT-qPCR analysis confirmed that DLG3 expression was decreased in ASD-NPCs (Figure 6B). Accordingly, the ASD risk gene DLG3 was selected for further analysis. To verify the relationship between miR-92a-2-5p and DLG3, we transfected miR-92a-2-5p inhibitor, miR-92a-2-5p mimic, scrambled control into NPCs, and then performed RT-qPCR. We observed that miR-92a-2-5p overexpression and downregulation significantly regulated DLG3 expression (Figure 6C). Taken together, these data suggested that DLG3 might be a target gene of miR-92a-2-5p.

## 4. Discussion

ASD is a class of heterogeneous neurodevelopmental disorders characterized by abnormal behaviors, which imposes a great burden on society and families. Although the exact etiology of ASD is currently unknown, abnormalities in neurogenesis and neurotrophic factor availability, as well as imbalance of E/I neurotransmission, have been hypothesized to be involved in the disorder [24,25]. Adult neurogenesis is a mechanism of structural plasticity whereby neurons are generated from NPCs exiting the cell cycle, migrating, and subsequently partially maturing into neurons in neurogenic niches in the mammalian (and human) brain [26,27]. Therefore, the balance between NPCs and neurogenesis must be precisely regulated, and lack of regulation may result in several neurological diseases [28,29]. NPCs derived from hiPSCs have been used to model neuropsychiatric disorders in previous studies, including ASD [30,31]. The transcriptome profiles from CON and ASD-NPCs were used to indicate neuronal tissue at early (4–10 weeks after conception) stages of prenatal brain development [32].

miRNAs are small noncoding RNAs and are involved in a variety of biological processes by regulating their target genes. Furthermore, miRNAs play essential roles in the brain, particularly in neuronal development and neurogenesis, and can be involved in ASD pathophysiology [33]. Vaccaro et al. investigated the expression of 26 candidate miRNAs from peripheral blood samples of ASD patients. Among these samples, miR-92a-2-5p was highly expressed in ASD blood compared with control samples [34].

Previously, cancer-related studies have shown that miR-92a can suppress cell proliferation and induce apoptosis [35,36]. Consistent with these findings, upregulation of miR-92a-2-5p could inhibit proliferation and enhance apoptosis of intestinal cells, indicating that miR-92a-2-5p may play an important role in the cell cycle, as well as in growth and differentiation [20]. MiR-92a-2-5p shares the same seed sequence with family members of the miR-17-92 cluster, which plays an important role in the central nervous system (CNS), implying that miR-92a-2-5p may also play a significant role in the CNS [37,38]. Aiming to identify the target genes of miR-92a-2-5p, we eventually narrowed the potential miR-92a-2-5p targets to DLG3. DLG3 is located at Xq13.1 and encodes the synapse-associated protein SAP102, which belongs to the membrane-associated guanylate kinase (MAGUK) protein family, extensively expressed in the brain [39]. DLG3 plays an important role in the signal pathway of synaptic plasticity. DLG3 mutations have been found in ASD patients, and have been identified as an ASD risk gene [40,41]. In this study, the expression level of DLG3 was inversely correlated with the expression of miR-92a-2-5p, implying that DLG3 may be a target gene of miR-92a-2-5p.

In the present study, we demonstrated that ASD-NPCs displayed delayed S phase progression accompanied by reduced proliferation, which may affect neuron differentiation. Consistently, ASD-NPCs showed reduced TUJ1^+^ and MAP2^+^ cell counts in the early differentiation stage. At the late stage of neuronal differentiation, inhibitory neurons and synapse were also decreased in ASD-NPCs. To investigate the specific role of miR-92a-2-5p in the course of ASD, we first revealed that miR-92a-2-5p was highly expressed in ASD-NPCs. Thereafter, the effect of miR-92a-2-5p downregulation on the proliferation and neuronal differentiation of ASD-NPCs was investigated. Knockdown of miR-92a-2-5p contributed to the improved phenotype of ASD-NPCs in terms of the prolonged S phase and proliferation. The neuronal differentiation deficiency of ASD-NPCs was also ameliorated by downregulation of miR-92a-2-5p, as indicated by the increased number of mature neurons and inhibitory neurons, suggesting its attractive therapeutic potential in treating ASD.

In conclusion, our study provides evidence that abnormal expression of miR-92a-2-5p directly contributes to the molecular pathogenesis of autism. Further research on the function of DLG3 in ASD-NPCs is needed, which can further explain the role of miR-92a-2-5p in ASD.

## Data Availability

Data are presented in the manuscript.

## Supplementary Materials

The following supporting information can be downloaded at: https://www.mdpi.com/article/10.3390/cimb44060166/s1, Figure S1: Gene ontology (GO) analysis for all genes predicted to be target genes of miR-92a-2-5p according to prediction algorithms. Table S1: Information of control and ASD patients. Table S2: Primer sequences for the qPCR experiment.
