# Supplementary material for: miR-92a-2-5p Regulates the Proliferation and Differentiation of ASD-Derived Neural Progenitor Cells
Source: Curr Issues Mol Biol. 2022 May 24;44(6):2431–42. doi: 10.3390/cimb44060166 (PMC9222067; doi:10.3390/cimb44060166)
Supplement: Supplementary file 1 [file cimb-44-00166-s001.zip › supplementary file.pdf]

Table S1. Information of control and ASD patients.

Individuals	Age	Sex	ASD	Stereotype behavior	Others	Medications
Patient 1	6	Male	+	+		—
Patient 2	7	Male	+	+	Sleep disturbance	—
Control 1	7	Male	—	—		—
Control 2	7	Male	—	—		—

Table S2. Primer sequence for RT-qPCR.

Gene	Primer sequences
hsa-miR-92a-2-5p	F: ACACTCCAGCTGGGGGGTGGGGATTTGTTGCATT<br>R: CTCAACTGGTGTCGTGGA
hCDK1	F: AAACTACAGGTCAAGTGGTAGCC<br>R: TCCTGCATAAGCACATCCTGA
hCDK4	F: ATGGCTACCTCTCGATATGAGC<br>R: CATTGGGGACTCTCACACTCT
hCDK2	F: CCAGGAGTTACTTCTATGCCTGA<br>R: TTCATCCAGGGGAGGTACAAC
hKi67	F: ACGCCTGGTTACTATCAAAAGG<br>R: CAGACCCATTTACTTGTGTTGGA
hcyclin A2	F: CGCTGGCGGTACTGAAGTC<br>R: CAGACCCATTTACTTGTGTTGGA
hcyclin B1	F: TTGGGGACATTGGTAACAAAGTC<br>R: ATAGGCTCAGGCGAAAGTTTTT
hcyclin E	F: ACTGAGCTGGGCAAATAGAGA<br>R: TCGCCATATACCGGTCAAAGA
hDLG3	F: AAGAGGTCCTTGTATGTCAGGG<br>R: CACCGATCTGCTCACTTTCTC
hFMAP2	F: CGAAGCGCCAATGGATTCC<br>R: TGAACTATCCTTGCAGACACCT

hSYP	F: CTCGGCTTTGTGAAGGTGCT<br>R: CTGAGGTCACTCTCGGTCTTG
hVGAT	F: TGCGACGACCTCGACTTTG<br>R: CGCTGATAATGGATGTCTCCCT
hGAPDH	F: AGGGCTGCTTTTAACTCTGGT<br>R: CCCCACTTGATTTTGGAGGGA
mmu-miR-92a-2-5p	F: ACACTCCAGCTGGGAGGTGGGGATTGGTGGCATT<br>R: CTCAACTGGTGTCGTGGA
mVGAT	F: ACCTCCGTGTCCAACAAGTC<br>R: CAAAGTCGAGATCGTCGCAGT
mMAP2	F: GCCAGCCTCAGAACAAACAG<br>R: AAGGTCTTGGGAGGGAAGAAC
mU6	F: GCTCGCTTCGGCAGCACA<br>R: AACGCTTCACGAATTTGCGTG
mGAPDH	F: AGGTCGGTGTGAACGGATTTG<br>R: TGTAGACCATGTAGTTGAGGTCA
