# Supplementary figures and images for: miR-92a-2-5p Regulates the Proliferation and Differentiation of ASD-Derived Neural Progenitor Cells
Source: Curr Issues Mol Biol. 2022 May 24;44(6):2431–42. doi: 10.3390/cimb44060166 (PMC9222067; doi:10.3390/cimb44060166)

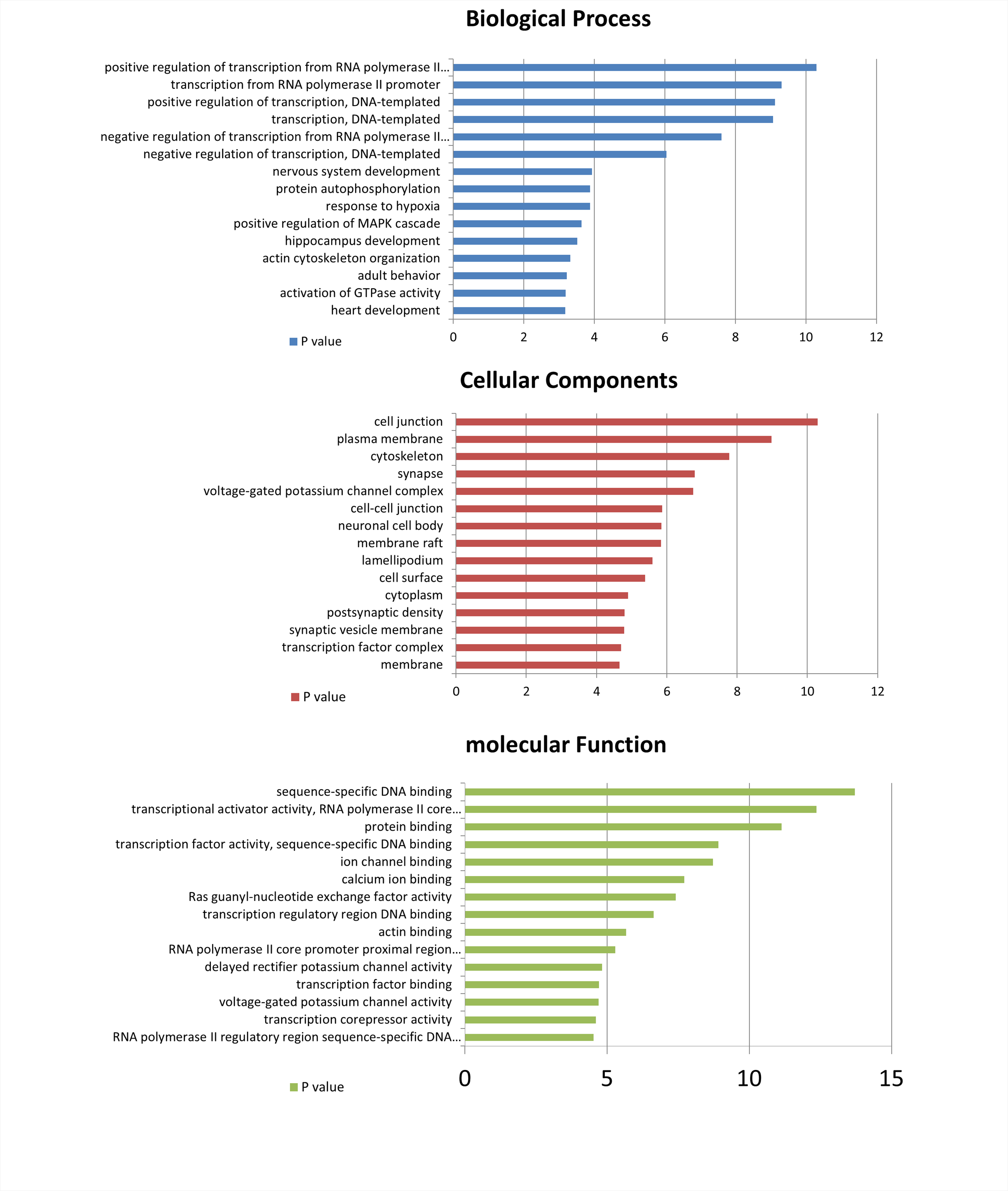

Supplement: Supplementary file 1 [file cimb-44-00166-s001.zip › Figure S1.tif]
